# Imaging of colorectal adenomas with pseudoinvasion and malignant polyps using two-photon excitation microscopy

**DOI:** 10.3389/fonc.2024.1394493

**Published:** 2024-06-14

**Authors:** Maria-Alexandra Florea, Lucian George Eftimie, Remus Relu Glogojeanu, Radu Hristu, George A. Stanciu, Mariana Costache

**Affiliations:** ^1^ Pathology Department, Central University Emergency Military Hospital, Bucharest, Romania; ^2^ Pathology Department, University of Medicine and Pharmacy, Carol Davila’, Bucharest, Romania; ^3^ Center for Microscopy-Microanalysis and Information Processing, National University of Science and Technology Politehnica Bucharest, Bucharest, Romania; ^4^ Department of Special Motricity and Medical Recovery, The National University of Physical Education and Sports, Bucharest, Romania

**Keywords:** pseudoinvasion, malignant polyps, multiphoton microscopy, second harmonic generation, collagen

## Abstract

**Introduction:**

Although the incidence and mortality rates of colorectal cancer exhibit significant variability, it remains one of the most prevalent cancers worldwide. Endeavors to prevent colorectal cancer development focus on detecting precursor lesions during colonoscopy. The diagnosis of endoscopically resected polyps relies on hematoxylin and eosin staining examination. For challenging cases like adenomatous polyps with epithelial misplacement, additional diagnostic methods could prove beneficial.

**Methods:**

This paper aims to underscore stromal changes observed in malignant polyps and polyps with pseudoinvasion, leveraging two-photon excitation microscopy (TPEM), a technique extensively employed in the medical field in recent years.

**Results and discussions:**

Both the subjective and quantitative analysis of TPEM images revealed distinct distributions and densities of collagen at the invasion front in malignant polyps compared to areas of pseudoinvasion. TPEM holds potential in discerning true invasion in malignant polyps from pseudoinvasion, offering enhanced visualization of local stromal changes.

## 1 Introduction

Colorectal cancer stands as the most prevalent malignancy affecting the gastrointestinal tract and ranks as the second leading cause of cancer-related mortality worldwide (1). Its incidence has been on the rise across both well-developed and medium to low-income countries in recent years, highlighting its significance as a global public health concern (2). Early detection of colorectal tumors is paramount in mitigating future morbidity and mortality rates.

Colorectal cancer presents a heterogeneous nature (3), both molecularly and morphologically. Most colorectal adenocarcinomas originate from precursor lesions, such as conventional adenomas or serrated polyps, which are typically marked by dysplasia (4). Colonoscopy emerges as the most effective method for detecting colonic polyps and cancers (5), aiming to identify and remove polyps with the potential to progress to invasive cancers. Advances in endoscopic techniques have significantly enhanced visualization of intestinal lesions and facilitated the removal of more complex lesions (6).

As per the WHO classification of digestive system tumors (2019), carcinoma *in situ* (Tis) denotes cancer cell invasion within the lamina propria, while T1 indicates invasion through the muscularis mucosae into the submucosa (7). Malignant polyps are characterized by tumoral cell invasion through the muscularis mucosa into the submucosa but not beyond it (8). The prevalence of malignant polyps among endoscopically removed polyps in screening programs can be as high as 11% (9). Features such as tubulovillous or villous architecture, size exceeding 10 mm, and high-grade dysplasia signify advanced adenomas and elevate the risk of malignant transformation (7).

Pseudoinvasion, also referred to as epithelial misplacement, is commonly observed in large pedunculated polyps, particularly in the sigmoid colon, resembling invasive carcinoma (10). It is characterized by the presence of dysplastic glands beneath the muscularis mucosa in the polyp head, stalk, or deeper regions. Unlike true infiltration, pseudoinvasion involves displaced adenomatous glands protruding through weakened areas of the muscularis mucosa, likely due to factors such as repeated stalk twisting, ischemia, or prior biopsy (11-12). Polyps exhibiting pseudoinvasion should be managed as ordinary adenomas, given their lack of malignant features.

The diagnosis of pseudoinvasion primarily relies on morphological examination, performed by an experienced pathologist. Deeper-level sections through the paraffin block and seeking a second opinion from a pathologist with expertise in digestive pathology are recommended for equivocal cases (13). Various methods, including immunohistochemistry - both epithelial (p53, E-chaderin) and stromal markers (collagen IV, MMP-1) and three-dimensional reconstruction or infrared spectroscopic techniques (12, 14), have been explored for the differential diagnosis of these entities. However, their utility in distinguishing challenging cases remains limited (15).

To highlight the local stromal alterations in both adenomas with epithelial misplacement and malignant polyps, we propose employing a nonlinear optical imaging technique. Specifically, we demonstrate the efficacy of two-photon excitation microscopy (TPEM) (16–19), a method that has gained prominence in clinical microscopy (20) in recent years and has been applied in endoscopic procedures (21, 22). TPEM simultaneously utilizes two nonlinear optical contrast mechanisms—second harmonic generation (SHG) and two-photon excitation fluorescence (TPEF)—to acquire images. SHG involves coherent optical second-order nonlinear effects (23), generating a new photon with double the energy of the initial photons upon interaction with non-centrosymmetric structures like collagen (24–28). TPEF microscopy (16), on the other hand, involves the simultaneous absorption of two photons with a total energy enough to produce a transition to an excited state. The subsequent spontaneous emission generates a photon with a slightly smaller energy than the total energy of the two excitation photons. It provides complementary information on cell and tissue morphology (29).

In this study, we employ both TPEF and SHG microscopy techniques to capture images from benign and malignant regions. Our aim is to compare collagen distribution patterns in normal tissue, benign polyps with pseudoinvasion, and malignant polyps at the invasive front.

## 2 Materials and methods

### 2.1 Sample preparation

The dataset utilized for this study comprises 14 polyps collected from 10 patients via polypectomy procedures conducted during colonoscopies and colectomies at the Central University Emergency Military Hospital (Bucharest, Romania). Nine polyps were identified as malignant, out of which five exhibited also pseudoinvasion. Additionally, there were four cases of adenomatous polyps showing pseudoinvasion, and one case of intramucosal adenocarcinoma. The samples were diagnosed by the Pathology Department and formalin-fixed paraffin-embedded (FFPE) blocks were stored in the histology laboratory at the Central University Emergency Military Hospital. Written informed consent was obtained from the patients and all samples were anonymized before analysis. All experiments were performed according to the relevant guidelines and regulations and in accordance with the Declaration of Helsinki. Samples obtained by cutting the FFPE blocks were sectioned (4–5 μm thick) and stained with hematoxylin and eosin (H&E). All samples were processed together in a single batch to reduce the impact of variations in histological stain recipes or procedures (30) that could lead to color discrepancies.

### 2.2 Microscopy techniques

All slides were scanned using a bright-field Aperio LV1 IVD Whole Slide Scanner (Leica Biosystems) equipped with a 20X objective lens. Expert pathologists identified regions of invasion and pseudoinvasion in malignant polyps, and areas of pseudoinvasion in adenomatous polyps. Following their annotations, these regions were further imaged by TPEM and compared qualitatively and quantitatively to identify collagen signatures that distinguish between the two types. Selected regions of interest (ROIs) were examined using a two-channel Leica TCS SP laser scanning confocal microscope configured for nonlinear imaging. The detailed description of the TPEM imaging system can be found elsewhere (31). The excitation source was a Ti:Sapphire laser (Coherent Chameleon Ultra II) tuned to a wavelength of 860 nm, with a pulse width of 140 fs and a repetition rate of 80 MHz. Laser power levels below 15 mW, as measured at the objective focus, were employed during scanning. SHG and TPEF signals were simultaneously collected in epi detection mode. A 10x magnification objective with a numerical aperture of 0.3 was employed to focus the excitation laser beam onto the samples and to collect TPEF and backward-generated SHG (BSHG) signals. The spectrally resolved detection setup inherent to the Leica TCS SP was utilized for capturing BSHG (425 to 435 nm) and TPEF (450 to 700 nm) signals on separate channels. Composite TPEM images were generated, with BSHG represented in the green channel and TPEF in the magenta channel. As the microscope objective’s maximum field-of-view is 1 x 1 mm^2^, image tiles were acquired and stitched together to create a mosaic covering a larger tissue area.

**Figure 1:**
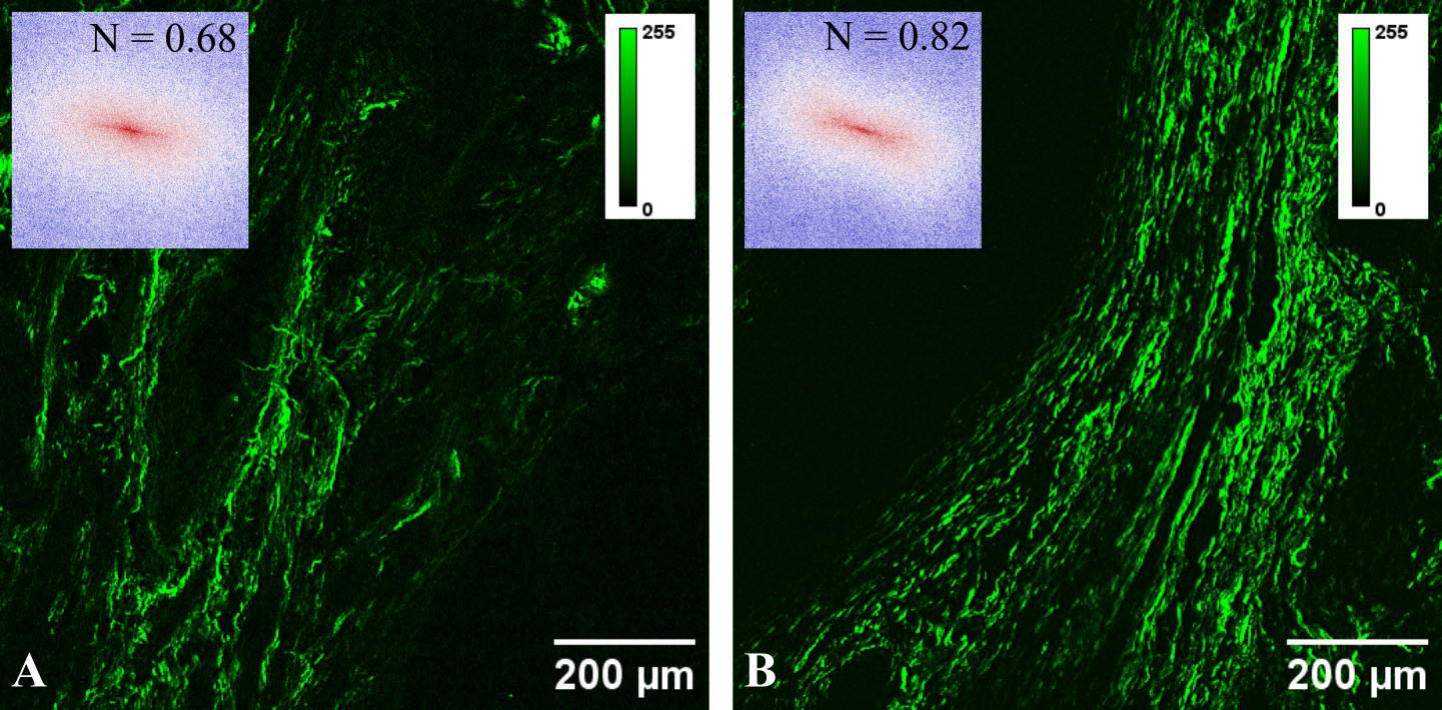
Typical SHG images for **(A)** pseudoinvasion and **(B)** invasion areas. The inset in each panel represents the FFT power spectrum and the corresponding collagen orientation index.

It is important to note that in H&E-stained tissue sections, the TPEF signal originates from regions containing eosin. Eosin binds nonspecifically to proteins within the cytoplasm, cell membrane borders, red blood cells, and extracellular structures. Conversely, the BSHG signal (**Figure 1**) originates from collagen fibers within the tissue, irrespective of the presence of stain.

### 2.3 Automated image analysis

Using the histogram analysis toolbox in ImageJ, four parameters associated with the distribution of pixel intensities in SHG images – Mean, Standard Deviation, Skewness, and Kurtosis – were calculated. While mean and standard deviation are well-recognized statistical moments, skewness and kurtosis characterize the distribution’s shape. To discern between collagen structures with distinct distributions, one additional parameter was considered. Pixels with SHG intensity surpassing a threshold were tallied, yielding the total collagen area ratio (TC-ratio), expressed as the ratio of this count to the total image area. Image thresholding was carried out using the automated thresholding toolbox in ImageJ, which encompasses 16 distinct thresholding methods. We visually examined how these algorithms worked on a set of TPEM images and found that the Triangle thresholding method provided the most effective results.

A second set of parameters, derived from the Gray-Level Co-Occurrence Matrix (GLCM), provides information about the spatial relationships between pixel intensities in an image. ImageJ’s GLCM texture plugin was used to compute the matrix for adjacent pixels in horizontal, vertical, and two diagonal directions. Information about collagen organization in SHG images can be extracted from the GLCM using the following parameters: Contrast, Homogeneity, Energy, Entropy and Correlation. Contrast and Homogeneity measure the local variations in an image, while Energy and Entropy can be used to give a quantitative measurement of the mutual orientation of collagen fibers (32). Correlation is based on the statistical analysis of pixel value dependence and can be used to assess periodicity within an image. Here, we employ a distance of one pixel between neighboring pixels, and we calculate the average values obtained from the horizontal, vertical, and two diagonal directions for each of the five parameters derived from the GLCM.

SHG images were utilized to assess collagen fiber orientation through FFT analysis with a custom-written ImageJ script. FFT power spectra images generated for each acquired SHG image (insets in **Figure 1**), were initially binarized and then fitted with an ellipse. A collagen orientation index (N) was computed based on the lengths of the minor (S) and major (L) axes of the ellipse: N = 1 − S/L (33). Consequently, collagen orientation can be expressed by an index ranging from 0 (indicating random fibers) to 1 (indicating aligned fibers).

An alternative approach to objectively quantify organization in an image involves fractal analysis. The SHG images underwent analysis after thresholding with the Triangle method, hence in a binary format, utilizing an ImageJ plugin (i.e., Fractal box count) to compute the fractal dimension.

For the statistical analysis, we employed the two-way unpaired Student’s t-test using Prism 10 (GraphPad Software, USA). Normality was evaluated using the D’Agostino and Pearson test. A p-value of 0.05 was used as the threshold for determining statistical significance in all tests.

## 3 Results

We examined a total of 14 colonic polyps, capturing whole slide images for each specimen. Additionally, corresponding TPEM images were acquired and analyzed for selected ROIs. Due to space constraints, only the most representative images are included in the manuscript. However, all images captured for this experiment, as well as high-resolution versions of the images presented here, are available in a public repository (DOI 10.17605/OSF.IO/JDRBN).

**Figure 2:**
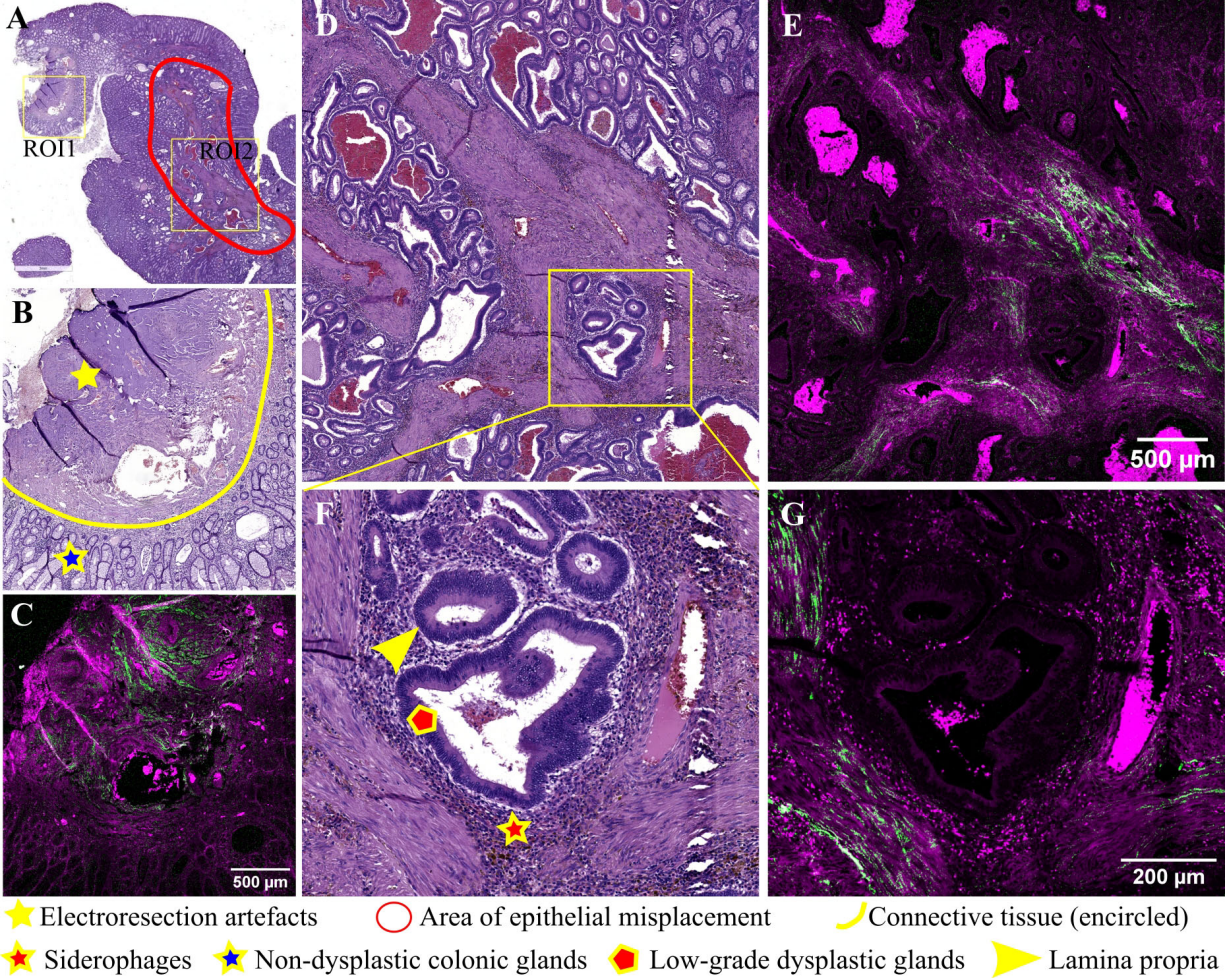
Adenomatous colonic polyp with pseudoinvasion area: **(A)** Large scale bright-field scanned image with rectangular ROIs selected for TPEM imaging; **(B)** Bright-field microscopy image of H&E-stained ROI1; **(C)** Corresponding TPEM image for ROI1; **(D)** Bright-field microscopy image of H&E-stained ROI2 corresponding to a pseudoinvasion area (low magnification); **(E)** Corresponding TPEM image for ROI2; **(F)** Bright-field microscopy image for a pseudoinvasion area – high magnification; **(G)** Corresponding TPEM image.

**Figure 2** displays typical images for an adenomatous polyp with low-grade dysplasia and epithelial misplacement. In **Figure 2A**, the lower half of the polyp, including the pedicle and implantation base, is depicted. **Figure 2B** illustrates a lateral margin of the pedicle, showcasing non-dysplastic glands and the underlying connective stroma, albeit with some artifacts induced by electro resection. Histologic examination utilizing overlaying BSHG and TPEF images reveals collagen fibers highlighted in the green channel, forming short and thin bands of various orientations. The magenta channel highlights non-dysplastic colonic glands with honeycomb arrangement and fibro-connective tissue from the base of the polyp (**Figure 2C**).

A pseudoinvasive area is identified in this polyp (**Figures 2D, F**), characterized by groups of dysplastic glands surrounded by lamina propria within the connective stroma of the polyp pedicle. Microhemorrhages and deposits of hemosiderin are observed near dysplastic glands. Surrounding misplaced glands, there is a diminished collagenous reaction, with short collagen fibers which lack consistent alignment, interspersed with smooth muscle cells (TPEM, **Figures 2E, G**).

**Figure 3:**
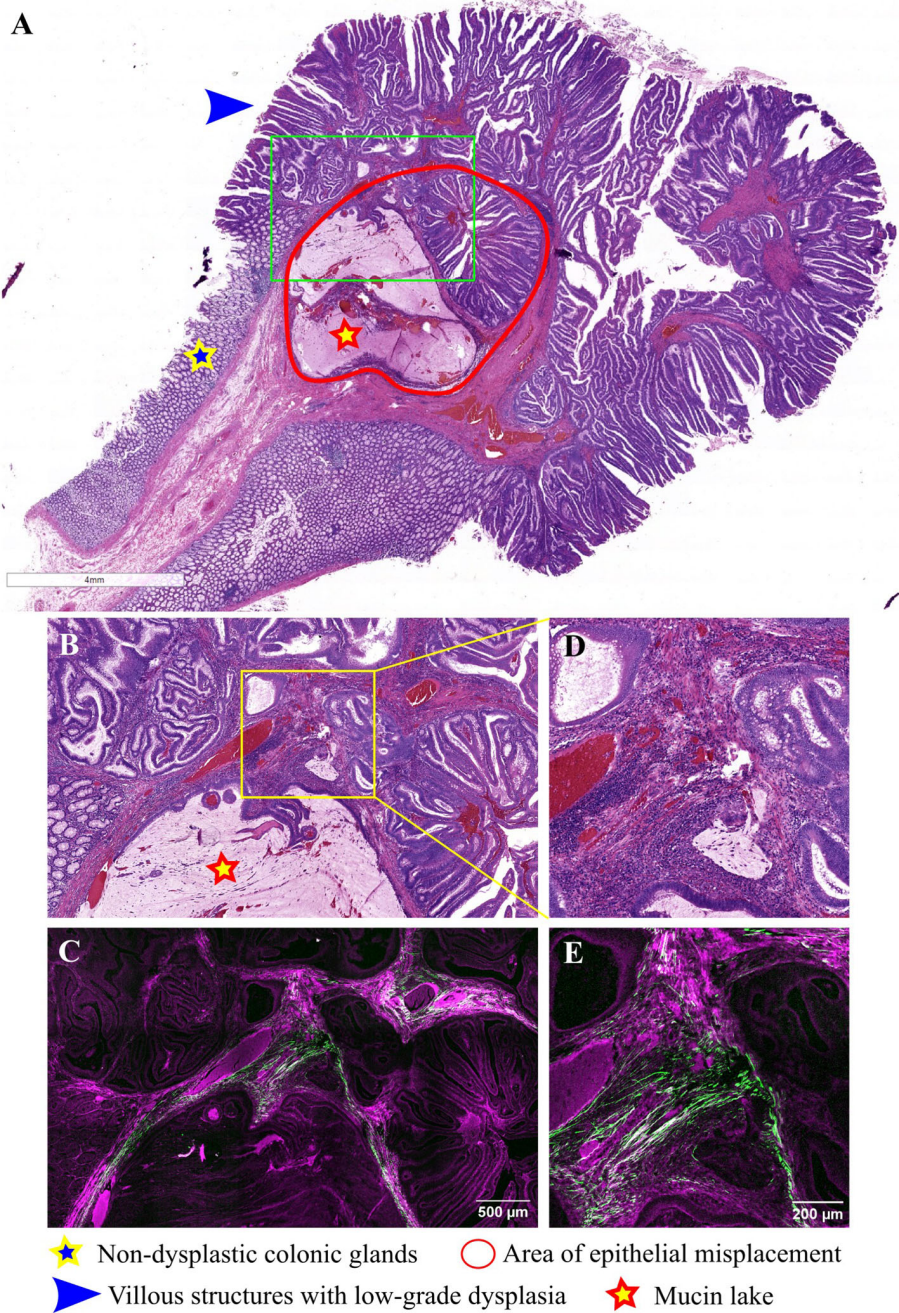
Pedunculated adenomatous colonic polyp with epithelial misplacement: **(A)** Large scale bright-field scanned image with a rectangular ROI selected for TPEM imaging; **(B)** Bright-field microscopy image of H&E-stained ROI corresponding to the area of epithelial misplacement (low magnification); **(C)** Corresponding TPEM image for the ROI; **(D)** Bright-field microscopy image of the area of epithelial misplacement (high magnification); **(E)** Corresponding TPEM image.

Similar observations are made in **Figure 3**, where a pedunculated adenomatous polyp with villous architecture, low-grade dysplasia, and an area of epithelial misplacement beneath the muscularis mucosae is depicted. It is composed of a rounded group of villous structures, along with two mucin lakes, with dysplastic epithelium at the periphery of the mucin lake, but without any floating tumoral cells and lacking a desmoplastic stroma (**Figures 3A, B, D**). TPEM images (**Figures 3C, E**) via the BSHG channel reveal a comparable distribution of collagen fibers as seen in the previous case.

**Figure 4:**
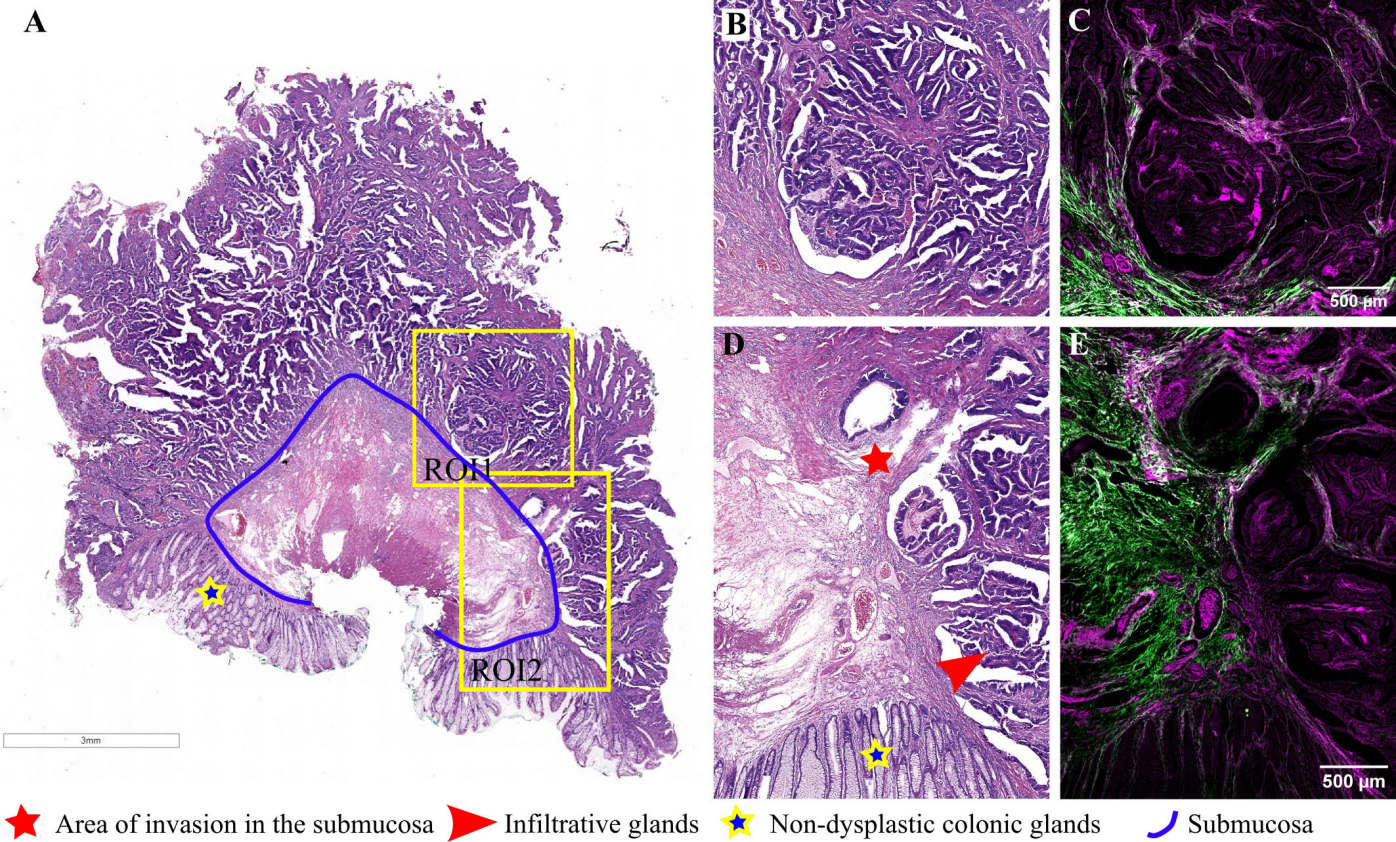
Adenocarcinoma invading the submucosa developed on a colonic adenomatous polyp: **(A)** Large scale bright-field scanned image with rectangular ROIs selected for TPEM imaging. **(B)** Bright-field microscopy image of H&E-stained tissue for ROI1; **(C)** Corresponding TPEM image for ROI1; **(D)** Bright-field microscopy image of H&E-stained tissue for ROI2; **(E)** Corresponding TPEM image for ROI2.

Representative images for another adenomatous polyp, displaying irregular, atypical glands infiltrating the submucosa and inducing a desmoplastic reaction, are presented in **Figure 4**. In this case, the diagnosis was adenocarcinoma arising in a tubulovillous adenoma, invasive in the submucosa. TPEM images (**Figures 4C, E**) show a more prominent fibrous reaction in the submucosa, with collagen fibers tending to accumulate more at the invasion front and less between invasive glands. At higher magnification, irregular and atypical glands infiltrating the submucosa, accompanied by a desmoplastic reaction, are depicted (**Figures 4B, D**). Collagen fibers at the invasive front appear organized in longer fascicles, with the collagen predominantly localized at the periphery of the tumor (TPEM, **Figures 4C, E**).

**Figure 5:**
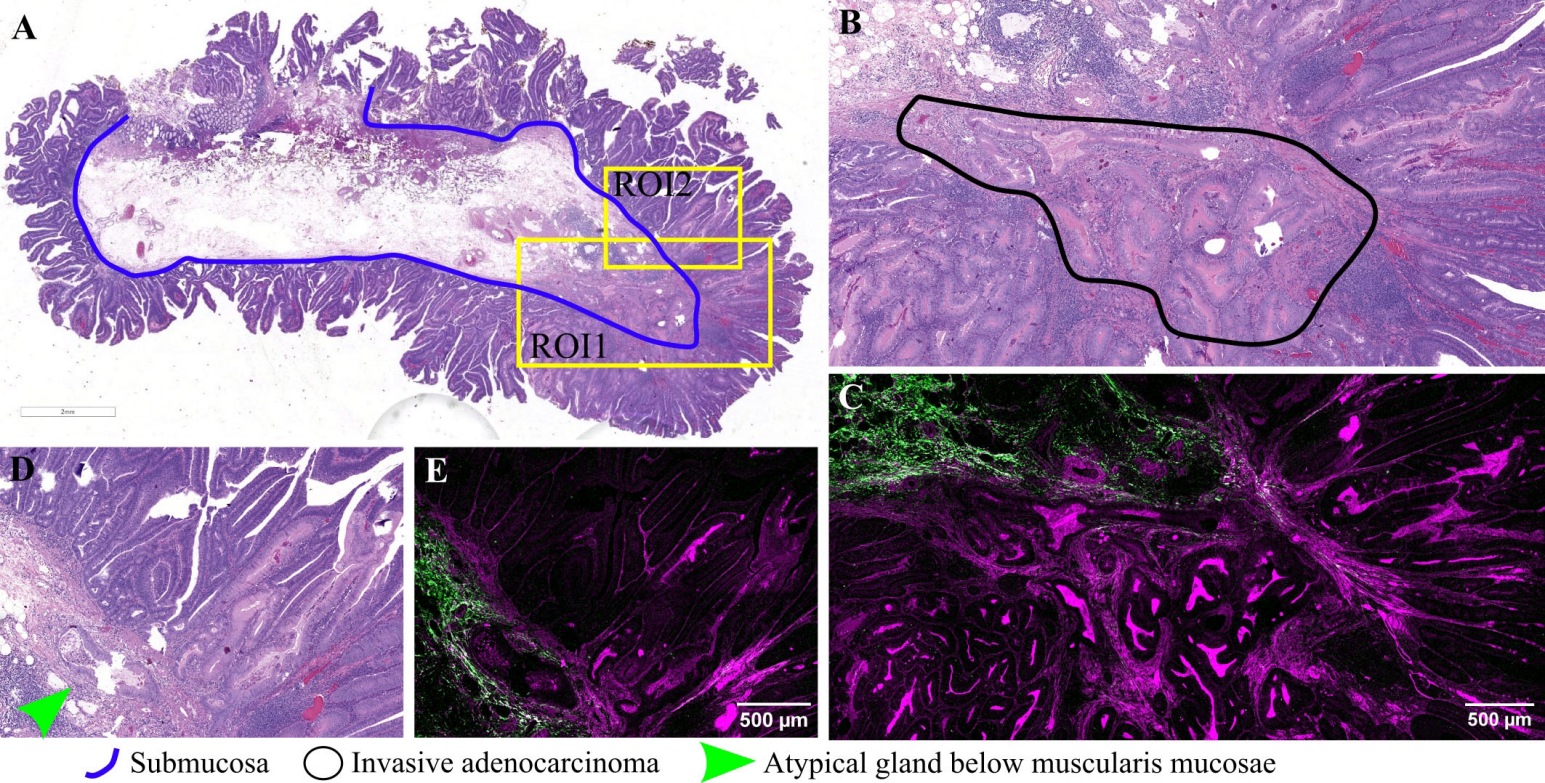
Adenocarcinoma arising in an adenomatous polyp: **(A)** Large scale bright-field scanned image with rectangular ROIs selected for TPEM imaging; **(B)** Bright-field microscopy image of H&E-stained tissue for ROI1; **(C)** TPEM image for ROI1; **(D)** Bright-field microscopy image of H&E-stained tissue for ROI2; **(E)** TPEM image for ROI2.

In the current study, another noteworthy polyp examined is a villous adenoma with an area of invasive adenocarcinoma, as evidenced by irregular, back-to-back glands infiltrating through the muscularis mucosae into the submucosa (**Figures 5A, B**). TPEM images reveal a prominent collagenous reaction surrounding malignant glands, characterized by longer fascicles of collagen fibers exhibiting a tendency to align parallel to the long axis of the tumor (**Figures 5C, E**).

**Figure 6:**
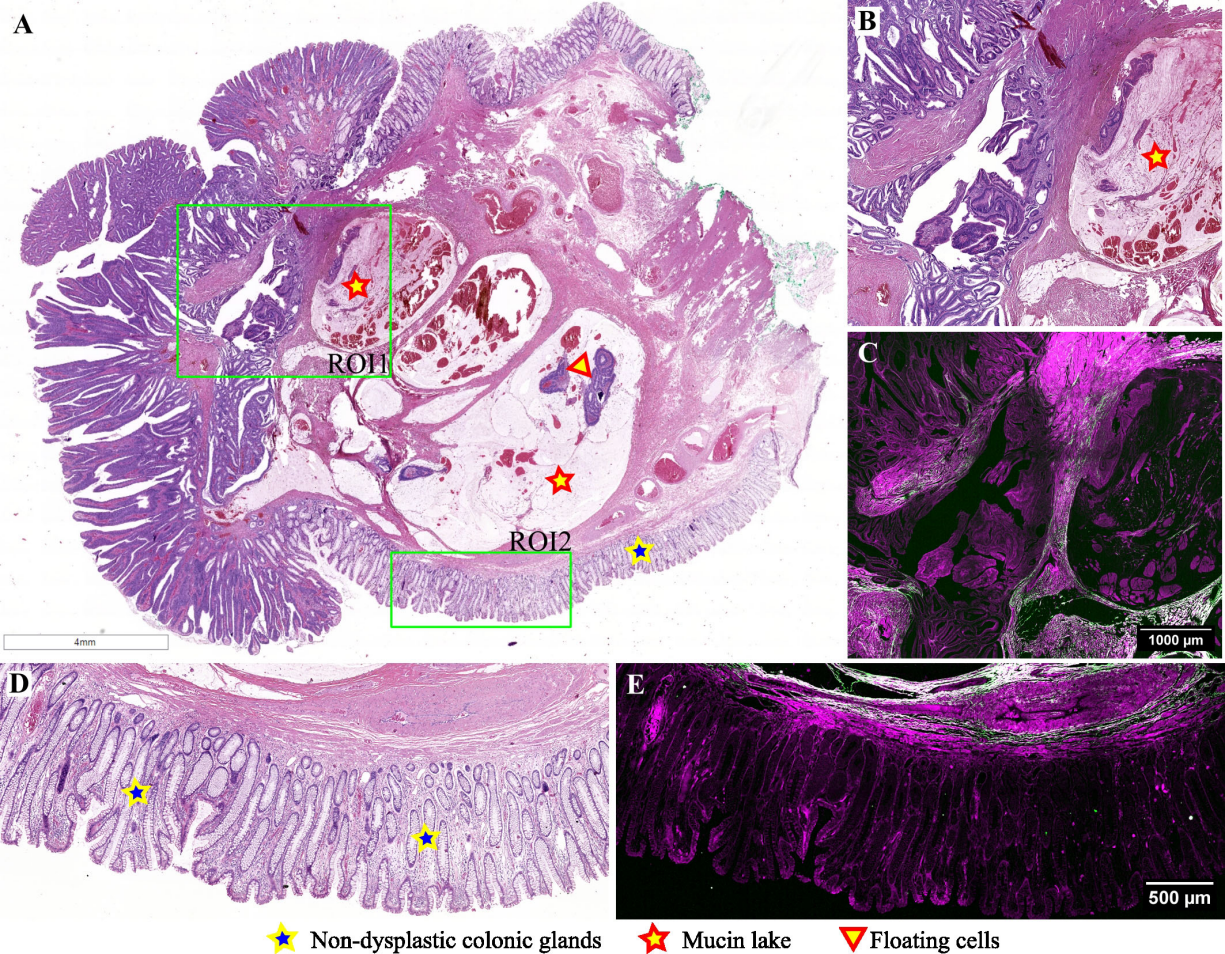
Adenomatous polyp with an area of invasive mucinous adenocarcinoma: **(A)** Large scale bright-field scanned image with rectangular ROIs selected for TPEM imaging; **(B)** Bright-field microscopy image of H&E-stained tissue for ROI1; **(C)** TPEM image for ROI1; **(D)** Bright-field microscopy image of H&E-stained tissue for ROI2, corresponding to healthy colonic tissue; **(E)** TPEM image for ROI2.

**Figure 6** presents an intriguing case of a pedunculated adenomatous polyp, wherein displaced atypical glands infiltrate through the muscularis mucosae, alongside mucin pools containing floating tumoral cells (**Figures 6A, B**). This case is diagnosed as mucinous adenocarcinoma arising in an adenomatous polyp because an area of invasive carcinoma was identified on another serial section from this polyp (not depicted in this image). An image selected from the lateral margins of this polyp depicts non-dysplastic colonic mucosa, muscularis mucosae, and a portion of a mucinous pool in the submucosa (**Figure 6D**). TPEM images illustrate the presence of a few short collagen fibers in the submucosa, below the muscularis mucosae in non-tumoral tissue (**Figure 6E**), along with a greater abundance of collagen fibers surrounding mucin lakes, arranged in longer fascicles.

As collagen features exhibited noticeable differences in the invasion and pseudoinvasion areas, we sought to assess whether quantitative parameters derived from TPEM images could differentiate between these regions. Since the visual examination of TPEM images returned differences in malignant polyps and polyps with pseudoinvasion which related to the collagen distribution outlined in the SHG images, the quantitative analysis was further performed only on the SHG channel in the TPEM images. For a comprehensive quantitative image analysis, we chose 88 images from invasion areas and 65 images from pseudoinvasion areas with significant collagen content from the acquired 1 x 1 mm² SHG image tiles. These image sets were then used to examine the distribution and organization of collagen in the SHG images using texture analysis methods detailed in the Methods section.

**Figure 7:**
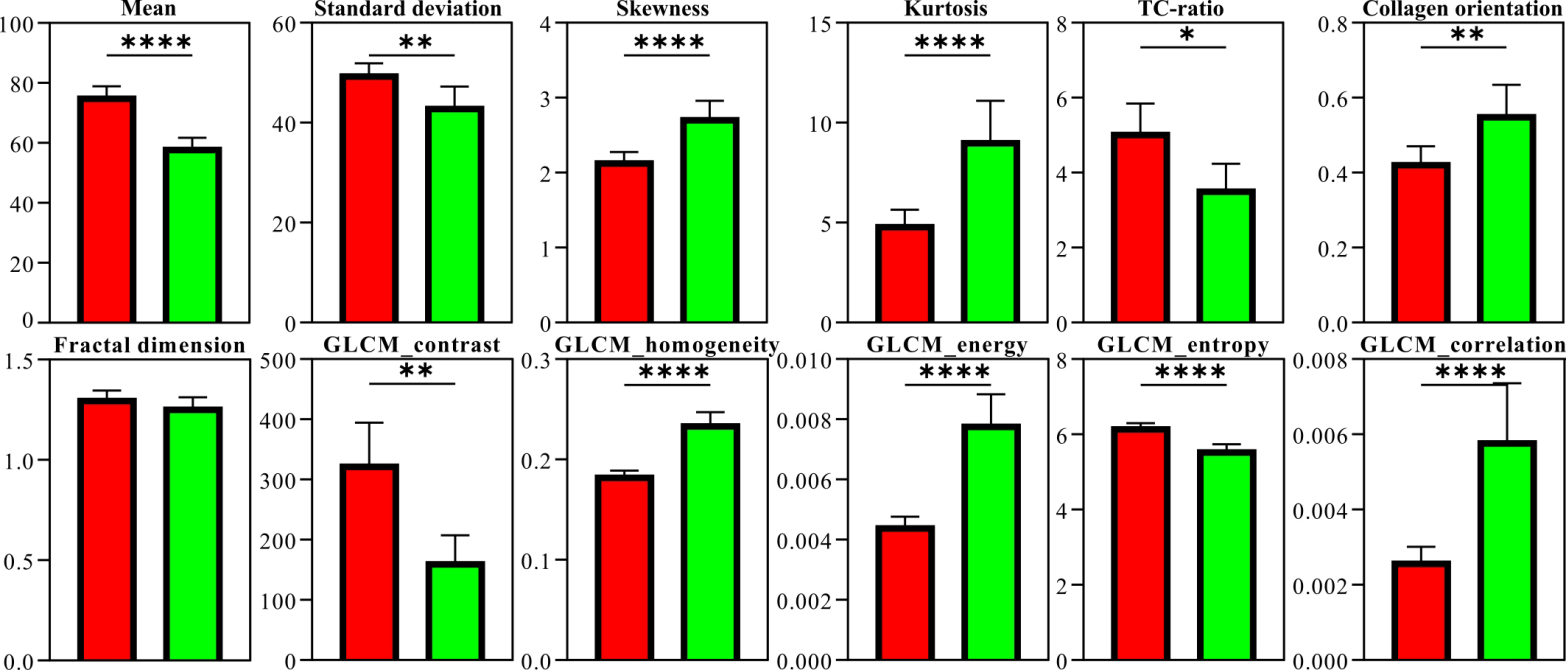
Parameters computed from SHG image sets for malignant polyps (red) and polyps with pseudoinvasion (green). The number of measurements was considered the number of image tiles for which the parameters were computed, hence 88 for invasion areas and 65 for pseudoinvasion areas. The vertical error bars represent the 95% confidence intervals (* p < 0.05, ** p < 0.01, **** p < 0.0001).

The results in **Figure 7** indicate that, except for one parameter (i.e., fractal dimension), all the computed parameters detected statistically significant differences between SHG images acquired on malignant polyps and polyps with pseudoinvasion.

## 4 Discussion

Differentiating between epithelial misplacement and true invasion in an adenomatous polyp presents a significant challenge, as it helps prevent the overdiagnosis of the lesion and unnecessary surgical interventions, thus impacting the patient’s quality of life significantly.

Histopathologically, malignant glands often exhibit irregular outlines, complex architecture (such as back-to-back glands, cribriform structures, or solid nests of atypical cells), intraluminal necrosis (dirty necrosis), and high-grade cytologic atypia—characterized by enlarged nuclei, hyperchromatism, prominent nucleoli, and atypical mitosis. The presence of desmoplasia, a stromal reaction surrounding cancerous cells, is a critical feature that, in the context of dysplasia, supports the diagnosis of invasive carcinoma. These histological changes are evident in our H&E-stained tissue samples from the polyps depicted in **Figures 4A, B, D**; **Figures 5A, B, D**; and **Figures 6A, B, D**.

We utilized a nonlinear optical method—two-photon microscopy—to elucidate local stromal changes in both malignant adenomas and adenomas with pseudoinvasion. The results are depicted as two-channel TPEM images, with SHG represented on the green channel and TPEF on the magenta channel. Relevant findings are derived from analyzing the SHG channel, which accentuates the collagen architecture in the examined samples. The samples imaged under the TPEM microscope were prepared for bright-field microscopy, hence the TPEF images contain nonspecific information collected on eosin fluorescence.

The desmoplastic reaction entails alterations in the extracellular matrix, a component vital for cellular communication, adhesion, and proliferation (34). This reaction was observed in three out of the five cases examined via TPEM (**Figures 4**, **5**, and **6C, E**), manifesting as an accumulation of collagen surrounding infiltrative glands. Collagen possesses properties such as fiber density, distribution, orientation, and organization, which serve as crucial markers in cancer research.

Conversely, pseudoinvasion is characterized by several histopathological features, including rounded displaced glands lacking infiltrative contours, surrounded by normal stroma of the lamina propria, alongside inflammatory cells and fragmented smooth muscle bundles exhibiting similar morphological changes to those observed in the surface epithelium (10). These specific pseudoinvasion changes were evident in the polyp depicted in **Figures 2D, F**, as well as **Figures 3B, D**, on H&E-stained sections. Notably, there is an absence of desmoplastic reaction around displaced glands, with inflammation, hemorrhage, and hemosiderin deposits observed in the stalk surrounding the glands. TPEM images highlighted a reduced presence of collagen fibers around pseudoinvasion areas, indicating a fibro-muscular reaction around prolapsed glands rather than true desmoplasia.

Displaced glands within the polyp may undergo dilation and accumulate mucus, unable to reach the lumen due to the entrapment of surrounding glands. Subsequently, these glands may rupture into the stalk, resulting in the formation of mucinous pools, which typically exhibit a rounded outline. In such cases, the epithelium is situated at the periphery of the mucinous pool rather than floating within it, as observed in invasive cancers (35). These distinctive features are discernible in the polyp depicted in **Figure 6**. Distinguishing invasion from epithelial misplacement in an adenomatous polyp with high-grade dysplasia poses a greater challenge (7). Isolated glands lacking surrounding lamina propria, poor differentiation, and evidence of vascular invasion lean towards a diagnosis of adenocarcinoma.

**Figure 8:**
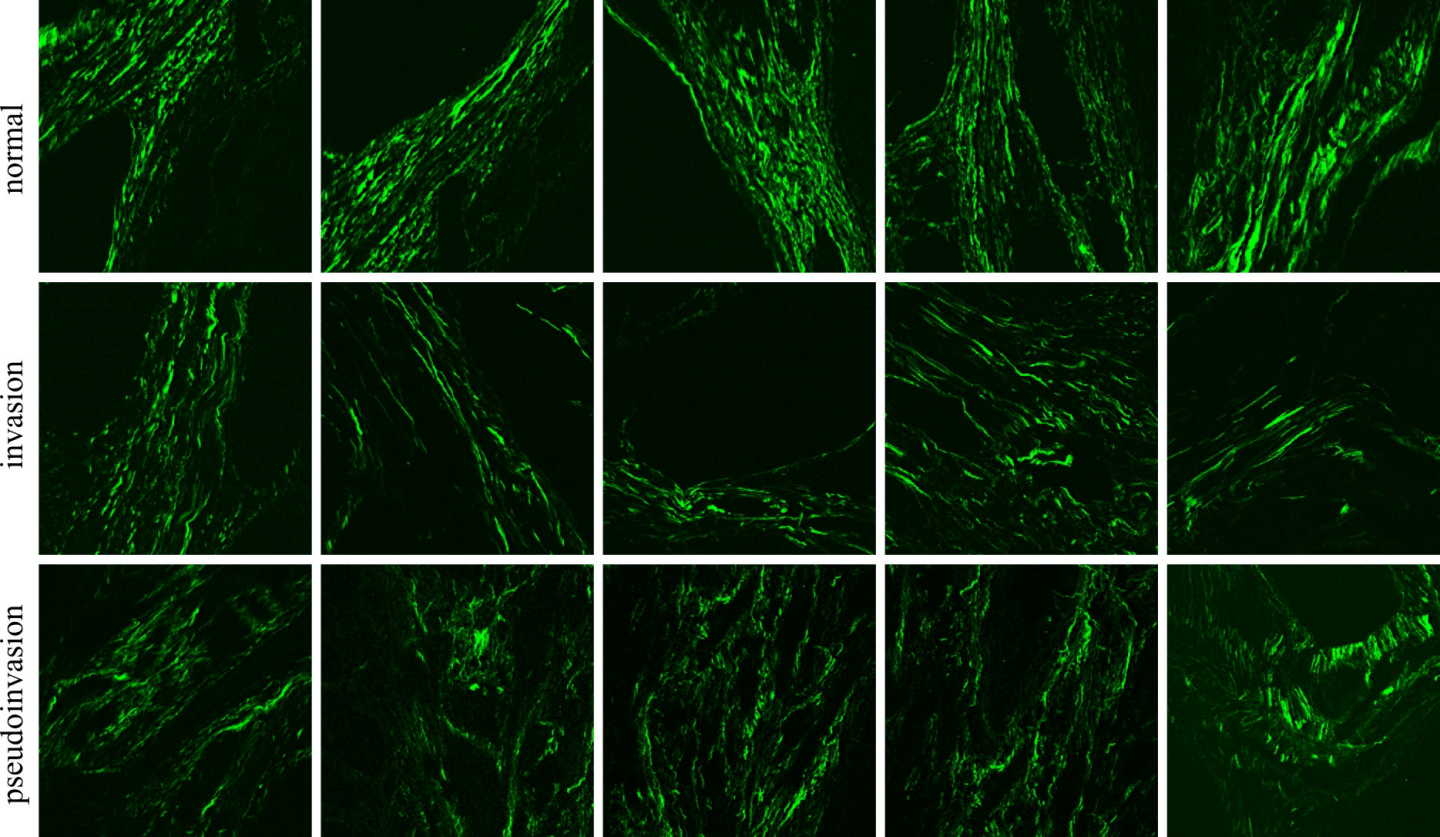
SHG images of normal, invasion and pseudoinvasion areas. Each image is 500 x 500 μm^2^.

Merely assessing an image obtained from an H&E-stained section makes it difficult to differentiate the desmoplastic stroma associated with submucosal invasion in malignant polyps from the fibro-muscular reaction present in polyps with pseudoinvasion. In both scenarios, collagen fibers tend to exhibit an eosinophilic compact appearance. However, our findings suggest that TPEM images, particularly the SHG images, offer superior visualization of collagen fibers compared to conventional microscopy. Analysis of collagen in the TPEM images unveils a distinct collagen architecture in the invasive area compared to regions of pseudoinvasion (**Figure 8**). Notably, our cases revealed more prominent collagen deposits in malignant polyps, particularly at the invasion front, exhibiting a unique organizational pattern compared to normal tissue from the base of an adenomatous polyp or areas of epithelial misplacement (pseudoinvasion). Such architectural changes imply a potential role of this reaction in impeding tumor extension.

The quantitative findings (**Figure 7**) can be partially associated with the qualitative observations from the TPEM images (**Figures 2**–**6**). The lower mean pixel intensities observed in polyps with pseudoinvasion, showing statistically significant differences compared to malignant polyps, suggest a reduced SHG signal. This reduction could stem from a decrease in collagen density, a decrease in the SH generation of individual collagen fibers, or a combination of both factors. To address this question, we calculated another parameter, namely TC-ratio, which assesses the total collagen area in an SHG image and specifically considers changes in collagen density. Notably, a statistically significant decrease in TC-ratio was also observed for polyps with pseudoinvasion. This outcome aligns with the notion of decreased collagen content in cases of pseudoinvasion.

The Standard Deviation of pixel intensities serves to measure the extent of dispersion within a dataset. Typically, a reduced standard deviation in the distribution of pixel intensities may indicate lower contrast, implying a more consistent image. In the case of polyps with pseudoinvasion, there is a statistically significant decrease in standard deviation compared to malignant polyps. This finding correlates with the diminished collagen content in polyps exhibiting pseudoinvasion.

As for Skewness, both distributions exhibit a positive skew, characterized by longer right tails. A higher skewness in pixel distributions within an image typically implies lower pixel values, thus resulting in a low-intensity (dark) SHG image (36). In our context, this may suggest either a reduced intensity of SHG signals from collagen or a decreased collagen density in the imaged region. The elevated Skewness value observed in polyps with pseudoinvasion aligns with the observation of lower collagen content.

Both distributions exhibit elevated positive Kurtosis. A statistically significant rise in Kurtosis has been noted in polyps with pseudoinvasion, signifying that pixel values are distributed in closer proximity to the mean compared to a normal distribution, with minimal variance attributed to infrequent extreme deviations. The lower Standard Deviation, associated with higher Kurtosis, indicates a narrower distribution of pixel values around the mean and suggests sharper features (collagen fibers) in the image.

While the previously discussed first-order statistical parameters, computed through the image histogram, are directly associated with the gray level distribution of pixel intensities, second-order statistical parameters for images are contingent on the spatial arrangements and correlation of pixel intensities.

Contrast is linked to the disparity between adjacent GLCM elements, measuring the uneven distribution of values within the GLCM. Elevated GLCM contrast signifies highly contrasting images, often associated with features distributed without a preferred alignment (37). A statistically significant increase in contrast was observed for malignant polyps compared to polyps with pseudoinvasion, possibly attributed to the presence of aligned collagen fibers in the former scenario. Homogeneity and contrast typically exhibit an inverse correlation, a trend that is consistent with our findings as well.

Energy identifies irregularities in textures, with a maximum value of one when the gray level distribution is consistent and uniform. Lower energy values arise when there is an increase in small entries in the GLCM, which may point to heterogeneous images. In our case, the results regarding energy and homogeneity suggest that the GLCM values exhibit a lower level of uniformity. Similar to Contrast and Homogeneity, Energy and Entropy also show an inverse correlation. A higher entropy value suggests a more complex texture in the image.

The findings from the GLCM analysis should be viewed in the context of the broader perception of an image, which extends beyond pixel intensity values to include factors such as context and surrounding patterns. This broader perspective can lead to varied visual interpretations, even when statistical measures suggest homogeneity. Although an image might exhibit high overall homogeneity, local variations or structures within it can make it visually compelling. For example, high homogeneity might stem from consistency across much of the image, while other areas may still display distinctive textures or patterns. Despite potential discrepancies between GLCM results and straightforward visual interpretations, these findings indicate changes in the collagen architecture between areas of true invasion and pseudoinvasion.

Correlation serves as an indicator of linear dependencies among gray levels in an image. Both malignant polyps and those with pseudoinvasions exhibited low Correlation values. A low Correlation typically signifies independent adjacent gray levels, indicating the absence of a significant regular pattern in the image.

Fractal analysis was conducted on binary images derived from image thresholding. Despite the prevalence of binary fractal analysis, we faced here one of its drawbacks, such as the requirement for image binarization through thresholding. In our case, no statistically significant differences were observed in the fractal dimension between malignant polyps and polyps with pseudoinvasions.

The collagen orientation index determined through FFT analysis exhibits a statistically significant elevated value for polyps with pseudoinvasion, indicating a more random distribution of collagen fibers compared to malignant polyps.

Previously, TPEM microscopy has been employed to characterize collagen alterations in various epithelial tumors, including breast, ovarian, gastric, colorectal, pancreatic, lung, bladder, thyroid, and skin cancer. Studies have revealed that as tumors progress, they tend to exhibit a higher quantity, lower organization, and increased linearity of collagen fibers (38). These findings align closely with the observations made in the two cases of malignant polyps described herein. However, in the context of colorectal pathology, TPEM has not been utilized for evaluating pseudoinvasion.

The quantitative imaging approach adopted in this study could pave the way for incorporating machine learning (ML) and deep learning (DL) techniques into colorectal cancer diagnosis (39) with significant implications for the advancement of TPEM technology. Over the past 20 years, TPEM has undergone significant development and has evolved to encompass diverse versions applicable in clinical settings (40). Despite its current advantages, TPEM has not achieved widespread adoption, partly due to challenges faced by end users, namely pathologists and surgeons, in interpreting the data. Consequently, they require retraining to effectively analyze TPEM images. ML and DL methodologies can automate the interpretation of TPEM images, assisting pathologists and surgeons in making more accurate and consistent diagnoses. ML and DL algorithms trained on large datasets of annotated TPEM and H&E-stained images could identify patterns and features indicative of colorectal cancer, potentially even discovering new markers not previously recognized by human experts. Moreover, the integration of ML and DL approaches with high-throughput TPEM systems capable of generating images of entire histological slides [e.g., widefield SHG microscopy (41, 42), and compact higher harmonic generation microscopy for unprocessed tissue (43)] will enable direct comparisons between H&E-stained slides and their TPEM counterparts (44). This integration could simplify TPEM images interpretation, rendering it a promising avenue for further exploration. It could also pave the way for personalized treatment plans based on the specific characteristics of each tumor, ultimately enhancing patient outcomes and revolutionizing colorectal cancer care.

By integrating TPEM microscopy with quantitative parameters derived from image texture analysis, including histogram analysis, gray level co-occurrence matrix, fractal analysis, and FFT analysis applied to SHG collagen images obtained from malignant polyps and polyps with pseudoinvasions, we have demonstrated the capability to distinguish between true invasion and pseudoinvasion sites based on collagen distribution in colonic polyps. Among the 12 parameters examined, 11 parameters exhibited statistically significant differences between the two considered classes. Our quantitative findings align with the observed alterations in collagen fiber organization, indicating a random organization in pseudoinvasion areas and a more structured distribution in true invasion sites.

## 5 Conclusions

Using two-photon excitation microscopy techniques, we identified significant qualitative and quantitative alterations in the ultrastructure of collagen within the two tumor types under investigation. There were significant differences in the orientation and quantity of collagen fibers between true invasion and pseudoinvasion in colonic polyps. Two-photon excitation microscopy demonstrated clear superiority in visualizing stromal changes when compared to conventional H&E images. Our research indicates that the integration of two-photon excitation microscopy with quantitative image analysis has the potential to distinguish true invasion in malignant polyps from pseudoinvasion, based on distinctive content and distribution of collagen fibers. This approach proves particularly valuable in cases presenting diagnostic challenges and may serve as a useful method in the early detection of colorectal cancer.

## Data availability statement

The datasets generated during and/or analyzed during the current study are available in a public repository (DOI: 10.17605/OSF.IO/JDRBN).

## Ethics statement

The studies involving humans were approved by Central University Emergency Military Hospital (Bucharest, Romania). The studies were conducted in accordance with the local legislation and institutional requirements. The participants provided their written informed consent to participate in this study.

## Author contributions

MF: Validation, Writing – review & editing, Writing – original draft, Methodology, Investigation. LE: Writing – review & editing, Visualization, Validation, Methodology. RG: Data curation, Writing – review & editing, Visualization, Investigation. RH: Visualization, Formal analysis, Writing – original draft, Validation, Investigation. GS: Writing – review & editing, Supervision, Project administration, Funding acquisition, Conceptualization. MC: Project administration, Writing – review & editing, Supervision.
